# Percutaneous Transluminal Mitral Commissurotomy in Pregnant Women with Severe Mitral Stenosis 

**Published:** 2019-01

**Authors:** Ata Firouzi, Niloufar Samiei, Somayyeh Ahmadi, Nasim Naderi, Parham Sadeghipour, Hamid Reza Sanati, Fahimeh Kashfi, Roya Sattarzadeh, Sedigheh Hantoushzadeh, Maryam Bayat, Sanaz Pourtaghi, Mohsen Nasiri

**Affiliations:** 1 Cardiovascular Intervention Research Center, Rajaie Cardiovascular, Medical, and Research Center, Iran University of Medical Sciences, Tehran, Iran.; 2 Heart Valve Diseases Research Center, Rajaie Cardiovascular, Medical, and Research Center, Iran University of Medical Sciences, Tehran, Iran.; 3 Rajaie Cardiovascular, Medical, and Research Center, Iran university of Medical Sciences, Tehran, Iran.; 4 Echocardiography Laboratory, Department of Cardiovascular Diseases, Imam Khomeini Hospital, Tehran University of Medical Sciences, Tehran, Iran.; 5 Maternal, Fetal and Neonatal Research Center, Valiasr Hospital, Tehran University of Medical Sciences, Tehran, Iran.

**Keywords:** *Heart valve diseases*, *Mitral valve stenosis*, *Balloon valvuloplasty*, *Pregnancy*

## Abstract

**Background: **Mitral stenosis tends to worsen during pregnancy because of the increase in the cardiac output and the heart rate. In nonresponders to medical therapy, percutaneous transluminal mitral commissurotomy (PTMC) may be performed when there is a suitable valvular anatomy. In this study, we aimed to investigate the clinical and fetal outcomes of pregnant women with mitral stenosis who underwent PTMC.

**Methods: **Thirty-one patients undergoing PTMC during pregnancy were enrolled in this study. The mitral valve area (MVA), the transmitral valve mean gradient (MVMG), and the severity of mitral regurgitation were assessed pre- and postprocedurally by transthoracic and transesophageal echocardiography. The radiation time was measured during the procedure. The patients were followed up during pregnancy, and the neonates were monitored for weight, height, the head circumference, the birth Apgar score, and the adverse effects of radiation for at least 12 months.

**Results: **PTMC was successfully performed on 29 (93.5%) patients. No maternal death or pulmonary edema was reported. The mean MVA significantly increased (from 0.73±0.17 cm^2^ to 1.28±0.24 cm^2^; P<0.001), and the mean MVMG significantly decreased (from 19.62±5.91 mmHg to 8.90±4.73 mmHg; P<0.001) after the procedure. A significant decrease in the systolic pulmonary artery pressure was also detected. Mitral regurgitation did not increase in severity in 16 (51.6%) patients. There was no significant relationship between the Apgar score, weight, height, and the head circumference at birth and at the radiation time.

**Conclusion: ** In our series, PTMC during pregnancy was a safe and effective procedure. Lowering the radiation time with low frame-count techniques confers a significant decrease in radiation-related complications.

## Introduction

Valvular heart diseases occur in 2% to 3% of the general population, with an increase in prevalence with advancing age,^1^ and complicate 0.5% to 1.5% of all pregnancies.^2^ Rheumatic heart diseases with isolated mitral stenosis are the dominant cardiac valvular problem in pregnancy.^3^ Importantly, mitral stenosis tends to worsen during pregnancy because of the increase in the cardiac output and the heart rate.^4^

The principal management for symptomatic patients with mitral stenosis during pregnancy concentrates on optimal medical therapy such as the use of beta-blockers and also bed rest. If medical management fails, balloon valvuloplasty in patients with a favorable anatomy might be considered a viable option.^4^ The high fetal and maternal mortality rate (1.8%–33%) of surgery and the acceptable procedural success of percutaneous transluminal mitral commissurotomy (PTMC) have made the latter modality a reliable therapy for pregnant women.^5^ Indubitably, PTMC during pregnancy remains a high-risk procedure. Apart from the procedural stress on the fetus and the potential hemodynamic deterioration during the intervention, radiation exposure is another major issue. Some of the major nonmalignant effects of ionizing radiation on the fetus or the neonate include growth and mental retardation, structural disorders, microcephaly, and seizure. These complications are not seen at doses lower than 50 mGy and are expected at doses of 100 mGy and higher.^6^ Pregnancy termination should be strongly recommended in case of radiation exposure above 10 rads.^7^

Our center is a tertiary cardiovascular center with long experience in performing PTMC. In this study, we sought to investigate the clinical and fetal outcomes of pregnant women with mitral stenosis who underwent PTMC.

## Methods

Between 2010 and 2016, all pregnant women with severe rheumatic mitral stenosis referred for PTMC to Rajaie Cardiovascular, Medical, and Research Center were included in the present study. The eligibility criteria for PTMC were comprised of second- or third-trimester pregnancy, severe rheumatic mitral stenosis, a mitral valve area (MVA) of equal to or smaller than 1 cm², dyspnea on exertion of the New York Heart Association Functional Classification (NYHA-FC) III or IV despite adequate medical therapy, severe hemoptysis, a history of acute pulmonary edema, a favorable anatomy according to the Wilkins criteria,^4^ mitral regurgitation (MR) of up to moderate severity on transesophageal echocardiography (TEE), and the absence of clots in the left atrium or the left atrial appendage on TEE.

The patients who did not fulfill the inclusion criteria for PTMC were excluded.

The study was approved by the Research and Ethics committee of Rajaie Cardiovascular, Medical, and Research Center, and informed consent was obtained from all the participants.

A comprehensive clinical history was taken to determine the symptoms and the NYHA-FC. Additionally, comprehensive physical, TEE, and transthoracic echocardiographic (TTE) examinations were performed by an expert echocardiographer. The MVA, the transmitral valve mean gradient (MVMG), and the severity of MR were determined in each subject in accordance with the recommendations of the American Society of Echocardiography.^8^

PTMC was performed by experienced interventional cardiologists via the Inoue technique.^9^ The balloon diameter for each case was chosen according to the patient’s body surface area. Appropriate shielding was used to protect the patient’s pelvis and abdomen during the procedure and, thus, limit fetal radiation exposure. The procedure was guided by TEE and a low frame-count fluoroscopy (7–8 frames per second) was selected in order to reduce radiation exposure. Radiation status was measured meticulously by a technician, who kept the chief operators informed at all times.

TTE was repeated 24 hours after the procedure to evaluate the efficacy of the procedure and the following indices: the MVA, the MVMG, the severity of MR, the systolic pulmonary artery pressure (SPAP), and the presence of pericardial effusion or iatrogenic atrial septal defects.

The main criterion for procedural success was defined as a decrease in the MVMG to at least half of the baseline (preprocedural).^4^

The maternal outcomes evaluated in the present study consisted of procedural success and the presence of maternal complications such as pericardial effusion, tamponade, death, severe MR, the need for surgical repair, and pulmonary edema. On the other hand, the fetal and child outcomes recorded in the present analysis comprised the presence of intrauterine growth retardation (IUGR), fetal loss, low birth weight, microcephaly, failure to thrive (defined as height growth below the third percentile for sex), any clinical malformation, and developmental delays including speech disorders.

Clinical follow-up after hospital discharge was accomplished by medical visits 1 month after the discharge, and the NYHA-FC of the patients was evaluated. After the first visit, the study population was followed up every 6 months in terms of maternal and child outcomes.

The newborns’ follow-up data were collected from the obstetric documents (for IUGR, the Apgar score, initial height, weight, and the head circumference) and the National Database for the Health of Newborns and Infants.

After successful PTMC procedures, 5 patients were lost to follow-up and were, therefore, analyzed as missing data.

For the statistical analyses, the Kolmogorov–Smirnov test was used to test normal distribution. The categorical variables were expressed as numbers and percentages and the quantitative variables as means±standard deviations (SDs). The paired sample *t* test was utilized for the parametric numeric data and the Wilcoxon test for the nonparametric numeric data. The correlations between the indices were estimated using the Pearson correlation. The IBM SPSS Statistics 19.0 for Windows (IBM Corp., Armonk, NY, USA) was used for all the statistical analyses. P values less than 0.05 were considered statistically significant.

## Results

The present study recruited 31 patients at a mean age of 31.81±4.32 years (min: 23 y, max: 41 y) and a mean gestational age of 22.91±6.12 weeks (min: 13 wk, max: 35 wk). Fifteen (48.4%) patients were in their second trimester of pregnancy, and the rest were in their third trimester. Table 1 depicts the demographic, clinical, and echocardiographic data for each patient at baseline.

All the patients had severe mitral stenosis (mean MVA=0.73±0.17 cm²) with a mean transvalvular pressure gradient of 19.62±5.91 mmHg.

The entire study population had a history of progressive dyspnea on exertion of the NYHA-FC III–IV despite medical therapy, significant hemoptysis, and/or pulmonary edema.

PTMC was performed successfully in 29 (93.5%) patients. The symptoms and impaired functional class were improved in all the patients who underwent successful PTMC procedures, and none of them experienced the NYHA-FC III or IV postprocedurally during the remaining period of pregnancy. No postprocedural acute decompensated heart failure (pulmonary edema) or maternal mortality was detected.

The mean fluoroscopic time during the study was 97.01±38.82 seconds. The main procedural findings are summarized in Table 2. The mean MVA rose by 0.55±0.11cm^2^, while the mean MVMG and the mean SPAP dropped by 10.74±4.01 mmHg and 25.62±15.41 mmHg, respectively.

The degrees of postprocedural MR were as follows: mild in 11 (35.5%) patients, mild to moderate in 13 (41.9%), moderate in 5 (16.1%), and moderate to severe in 2 (6.5%). Table 3 illustrates the pre- and postprocedural MR severity in the studied population.

The degrees of MR severity did not increase in more than half of the patients (16 cases [51.6%]). However, there was a statistically significant rise in MR severity after the procedure (P<0.001).

**Table 1:** Baseline characteristics of the study population*

Maternal age (y)	31.81±4.32
Gestational age (wk)	22.91±6.12
Mean MVA before PTMC (cm²)	0.71±0.23
Mean transvalvular pressure gradient before PTMC (mmHg)	19.62±5.91

MVA, Mitral valve area; PTMC, Percutaneous transluminal mitral commissurotomy

* Data are presented as mean±SD

**Table 2:** Main procedural outcomes of the 31 pregnant women undergoing PTMC

Procedural Index	Before PTMC	After PTMC	P
Mean MVA (cm^2^)	0.73±0.17	1.28±0.24	<0.001
Mean MVMG (mmHg)	19.62±5.91	8.90±4.73	<0.001
SPAP (mmHg)	73.03±22.8	47.4±13.60	<0.001

PTMC, Percutaneous transluminal mitral commissurotomy; MVA, Mitral valve area; MVMG, Mitral valve mean gradient; SPAP, Systolic pulmonary artery pressure

**Table 3:** MR severity before and after PTMC

MR Severity	Before PTMC		After PTMC	
	Frequency	Percentage	Frequency	Percentage
Trivial	4	12.9	-	-
Mild	19	61.3	11	35.5
Mild to moderate	6	19.4	13	41.9
Moderate	2	6.5	5	16.1
Moderate to severe	-	-	2	6.5

MR, Mitral regurgitation; PTMC, Percutaneous transluminal mitral commissurotomy

Two (6.5%) procedures resulted in pericardial effusion and tamponade; both of the patients, therefore, underwent closed pericardiocentesis. Ultimately, they were discharged alive in a good clinical condition.

Two iatrogenic small (5 mm) atrial septal defects were detected on TEE within 24 hours of the procedure. These 2 defects (6.5%) did not have any relationship with the radiation time (P=0.251).

There were 3 (11.1%) preterm labors. The etiology was identified in 2 patients: gestational diabetes mellitus and premature rupture of membranes. None of the preterm labors occurred periprocedurally.

Two patients underwent induced abortions. In one of them, PTMC was complicated by the chordal rupture of the MV, necessitating an induced abortion followed by mitral valve replacement (MVR). This was the only patient in the whole study to need surgery. The other patient continued to suffer from severe pulmonary hypertension despite a successful procedure and a significant drop in the SPAP early after the procedure, which was an indication for an induced abortion.

There was 1 (3.2%) case with stillbirth due to IUGR. Twin pregnancies were reported in 3 women, for whom the data were analyzed as a mean of twins.

As was described previously, in this case series, only 1 patient was candidated for surgical MVR due to chordal rupture. In addition, 5 patients failed to participate in the follow-up and 4 pregnancies did not lead to a live neonate. Therefore, there were 25 neonates and since 3 pregnancies resulted in twins, there were 22 patients for the final analysis.

Birth weights were between 1500 gr and 3600 gr, with a mean of 2800.12±592.39 gr. There was no relationship between weight at birth and gestational age (P=0.971) or the radiation time (P=0.197).

A low birth weight was reported in 9 (33.3%) neonates, with 5 of the mothers having undergone PTMC in the second trimester and the other 4 in their third trimester. There was no significant relationship between a low birth weight and gestational age (P=0.601) or the radiation time (P=0.576).

Height at birth was between 45 and 53 cm, with a mean of 49.04±2.39 cm. The head circumference at birth was between 32 and 38 cm, with a mean of 34.28±1.67 cm. There was no significant relationship between the head circumference at birth and the radiation dose (P=0.165) or gestational age (P=0.482).

The Apgar score of the second minute after birth was 8 in 4 (19%) neonates and 9 to 10 (81%) in the rest. In 2 of these 4 cases with a lower Apgar score, PTMC had been performed on the mothers in the second trimester. In these 4 newborns with a lower Apgar score, the time of radiation was 100, 65, 60, and 70 seconds and there was no significant relationship between the Apgar score at birth and the radiation time (P=0.191).

Weight growth in the first year was below the third percentile for sex in 2 (9.1%) cases, between the third and the 15th percentiles in 1 (4.5%), between the 50th and the 85th percentiles in 5 (22.7%), between the 85th and the 97th percentiles in 12 (54.5%), and above the 97th percentile in 2 (9.1%). There was no significant relationship between weight growth and the radiation dose (P=0.615) or gestational age (P=0.918).

Height growth in the first year was below the third percentile for sex in 2 (9.1%) cases, between the third and the 15th percentiles in 2 (9.1%), between the 50th and the 85th percentiles in 3 (13.6%), between the 85th and the 97th percentiles in 10 (41.5%), and above the 97th percentile in 5 (22.7%).

The growth curve for the head circumference in the first year was below the third percentile for sex (i.e., microcephaly) in 1 (4.5%) case, between the third and the 15th percentiles in 1 (4.5%), between the 15th and the 50th percentiles in 2 (9.1%), between the 50th and the 85th percentiles in 5 (22.7%), between the 85th and the 97th percentiles in 11 (50%), and above the 97th percentile in 2 (9.1%). The only case of microcephaly was a twin pregnancy and, importantly, both twins showed failure to thrive and the head circumference as well as the other growth parameters did not improve to 18 months of life. There was no significant relationship between the prevalence of microcephaly and gestational age (P=1.001) or the radiation time (P=0.391). Two children showed failure to thrive according to the growth curve (9.1%); one of them was a twin. The rest of the children had a normal development over a 12-month period, and none of them developed delays in speech and/or seizure.

## Discussion

In the present study, we present a report on the outcome of PTMC in the pregnant women referred to our center. Our series consisted of 31 pregnant women who underwent PTMC, with a success rate of 93.5%. The mean fluoroscopic time in our study was 97.01±38.82 seconds, which is approximately 4.5 times shorter than that in other similar studies. In our patients, the MVA, the MMVG, and the SPAP decreased significantly. In addition, in all the patients with successful PTMC procedures, the symptoms of heart failure were completely resolved. During the study, no mortality was encountered.

Over the past decade, hospital mortality related to PTMC in pregnant women has declined to less than 1%, with procedural success rates reported to be as high as 89% to 100%. The majority of these patients were between 20 and 30 years of age and in their second trimester of pregnancy at the time of PTMC.^7^ On average, catheter balloon commissurotomy increases the MVA to 2.0 cm².^10^^-^^14^ Several studies on the role of PTMC in pregnant women have reported a twofold increase in the postprocedural MVA (0.8–1 to 1.2–2.5).^5^^, ^^10^^, ^^12^^, ^^15^^-^^20^ In the majority of these studies, the MVA was measured using the Gorlin equation. However, in recent works, including our study, the MVA was measured via the direct planimetry method using TTE. In a study by Ben Farhat et al.,^12^ the mean MVA by planimetry increased from 1.07 cm² to 2.32 cm², which is slightly higher than that in our study (0.73±0.17 cm² to 1.28±0.24 cm^2^). The mean MVMG in almost all the relevant studies decreased to one-third of the procedural value, except in the study by Ben Farhat, who reported a decrease in the MVMG from 27 mmHg to 5 mmHg. In our study, the MMVG exhibited a significant decrease (from 19.64±5.96 mmHg to 8.90±4.73 mmHg; P<0.001). Additionally, the SPAP significantly decreased in our series (from 73.03±22.8 mmHg to 47.4±13.6 mmHg; P<0.0001). Similarly, Salehi et al.^20^ in Madani Center in the Iranian city of Tabriz, reported a drop from 58 mmHg to 38 mmHg in the SPAP. Our slightly lower MVA might be explained by 2 reasons: firstly, in comparison with other studies, our patients had a significantly low MVA before the treatment; and secondly, our main goal was to create a bridge therapy with the least possible radiation dose, by which both mother and fetus would have a safe pregnancy course. An MVA higher than 2 cm^2^ would be ideal but not necessary.

The mean fluoroscopy time was between 5.5 and 9 minutes in several studies,^5^^, ^^10^^-^^12^ while in our study, it was approximately 4.5 times shorter (i.e., 97.01±38.82 sec), which is below the harmful radiation time. During catheter balloon commissurotomy, the radiation exposure to the fetus is calculated to be less than 0.2 rad with the use of abdominal and pelvic shielding. Adverse effects on the fetus are reported to occur with radiation exposures of more than 5 rads.^7^ The shorter duration of radiation in our center may be due to its high number of PTMC procedures, which has significantly contributed to the experience of the interventional cardiologists.

In our case series, we had a 93.5% procedural success rate and the symptoms of heart failure were improved in all of our patients with successful PTMC procedures. Moreover, none of our patients experienced the NYHA-FC III or IV after the procedure or during the rest of their pregnancy. A study by Steves et al.^15^ on 71 pregnant women with severe symptoms who underwent PTMC showed a 100% success rate, with 98% of the patients reaching the NYHA-FC I-II. A similarly rapid relief of symptoms was reported by Patel et al.^16^ It is deserving of note that PTMC has a positive effect on the quality of life, with symptomatic improvements observed in 90% of cases in the immediate post-commissurotomy period; this improvement in the hemodynamic profile leads to a marked clinical improvement through the remainder of pregnancy.^12^ Fortunately, in the majority of the previous studies, the relief of symptoms conferred by PTMC has been accompanied by considerably low major complications (ranging from 0 to 9%), and it continues to decline as expertise in the procedure improves with time.^18^ In the present work, 2 patients were complicated by tamponade and 1 was candidated for surgical MVR due to chordal rupture. Also in 2 patients, non-symptomatic iatrogenic atrial septal defects were detected on follow-up imaging. Crucially, no maternal mortality occurred in the present study. Previous research has demonstrated a low risk of postprocedural MR requiring surgical interventions (0–5%) and maternal mortality (0.2%).^7^ In the Norrad and Salehian^21^ case series of 71 pregnant women, there was a 0.2% rate of maternal mortality, and 4.6% of the study population needed urgent MVR because of severe MR. However, in the study by Ben Farhat^12^ on 44 pregnant women, no maternal or fetal death was seen and 1 procedure resulted in severe MR. Elsewhere, Routray et al.^11^ reported 1 case of tamponade and 1 stillbirth.^11^

No maternal or acute decompensated heart failure (pulmonary edema) was seen in our group of patients, and only 1 patient was emergently scheduled for surgical MVR due to chordal rupture. The rate of maternal death and major cardiac events and abortion caused by complications was also very low in other studies.^5^^, ^^9^^, ^^16^^, ^^20^

In our case series, we found 4 preterm labors, 5 pregnancies with low birth weights, 3 abortions, and 1 stillbirth due to IUGR, which appears to be acceptable in comparison with other studies. Steves et al.^15^ reported a 13% rate of preterm labor in their study. In an investigation by Abdi et al.,^5^ no abortion was detected and 1 stillbirth was reported. Routray and colleagues,^11^ in their analysis of 40 pregnant women who underwent PTMC, found no maternal death, abortion, or IUGR.

We found no significant relationships between the prevalence of microcephaly, low birth weight, the Apgar score, growth, failure to thrive, and gestational age and the radiation time. All the neonates had a normal development over our 12-month follow-up period. In addition, in the latter stages, none of the newborns were complicated by delays in speech, seizure, or cancer. Abdi et al.^5^ reported no clinical abnormality in the newborns experiencing PTMC in their study. Steves et al.^15^ reported a normal birth weight and a normal growth in 44 months of follow-up in their investigation. Similar results were reported by Ben Farhat et al.^12^ in their 12 months of follow-up. Among the relevant studies, the longest (17 y) follow-up was done by Gulraze et al.,^18^ who detected a normal growth in all their live neonates. In that large case series, nevertheless, the authors reported 1 stillbirth resulting from obstetric causes, 1 case of sudden infant death syndrome, and 1 infantile death at 8 months due to pneumonia as infantile mortality.

## Conclusion

Our case series showed the safety and efficacy of PTMC in pregnant women. Notably, with a considerably lower radiation time, we succeeded in attaining good maternal and fetal outcomes by comparison with other studies. Therefore, in case of severe and critical mitral stenosis during pregnancy, PTMC might be considered a valuable bridging therapy with a view to protecting both mother and fetus.

## Notes:

***This paper should be cited as: ***Firouzi A, Samiei N, Ahmadi S, Naderi N, Sadeghipour P, Sanati HR, Kashfi F, Sattarzadeh R, Hantoushzadeh S, Bayat M, Pourtaghi S, Nasiri M. Percutaneous Transluminal Mitral Commissurotomy in Pregnant Women with Severe Mitral Stenosis. J Teh Univ Heart Ctr 2019;14(1):12-17.
